# Comparison of Deep-Water Viromes from the Atlantic Ocean and the Mediterranean Sea

**DOI:** 10.1371/journal.pone.0100600

**Published:** 2014-06-24

**Authors:** Christian Winter, Juan A. L. Garcia, Markus G. Weinbauer, Michael S. DuBow, Gerhard J. Herndl

**Affiliations:** 1 Department of Limnology and Oceanography, University of Vienna, Vienna, Austria; 2 Observatoire océanologique, Sorbonne Universités, Villefranche/mer, France; 3 Observatoire océanologique, CNRS UMR 7093, Villefranche/mer, France; 4 Institut de Génétique et Microbiologie, Université Paris-Sud, Orsay, France; 5 Department of Biological Oceanography, Royal Netherlands Institute for Sea Research, Den Burg, The Netherlands; Universidade Federal do Rio de Janeiro, Brazil

## Abstract

The aim of this study was to compare the composition of two deep-sea viral communities obtained from the Romanche Fracture Zone in the Atlantic Ocean (collected at 5200 m depth) and the southwest Mediterranean Sea (from 2400 m depth) using a pyro-sequencing approach. The results are based on 18.7% and 6.9% of the sequences obtained from the Atlantic Ocean and the Mediterranean Sea, respectively, with hits to genomes in the non-redundant viral RefSeq database. The identifiable richness and relative abundance in both viromes were dominated by archaeal and bacterial viruses accounting for 92.3% of the relative abundance in the Atlantic Ocean and for 83.6% in the Mediterranean Sea. Despite characteristic differences in hydrographic features between the sampling sites in the Atlantic Ocean and the Mediterranean Sea, 440 virus genomes were found in both viromes. An additional 431 virus genomes were identified in the Atlantic Ocean and 75 virus genomes were only found in the Mediterranean Sea. The results indicate that the rather contrasting deep-sea environments of the Atlantic Ocean and the Mediterranean Sea share a common core set of virus types constituting the majority of both virus communities in terms of relative abundance (Atlantic Ocean: 81.4%; Mediterranean Sea: 88.7%).

## Introduction

Generally, the deep ocean is characterized by the absence of light, high pressure, low temperature, high inorganic nutrient concentrations, and a dominance of recalcitrant organic matter [1]. Also, prokaryotes (here used to denote members of the phylogenetic domains *Bacteria* and *Archaea*; no phylogenetic relationship is implied), viruses, and other planktonic single-celled organisms decrease in abundance with increasing depth [2]. However, the deep Mediterranean Sea differs from the global ocean, as water temperatures at depth remain at ∼13°C year round. The high temperatures at depth are due to the high salinity of Mediterranean Sea waters, allowing deep vertical mixing driven by winter storms during the non-stratified period [3]. As a consequence, prokaryotic and viral communities in deep waters of the Mediterranean Sea display seasonality and are as dynamic as in the surface waters [4], [5], [6].

Viruses, especially those infecting prokaryotes, are the most abundant biological entities in marine environments and enhance dissolved organic matter cycling due to lysis of their prokaryotic host cells [7], [8], [9]. Viruses are also considered to be an important factor in the evolution of prokaryotic communities in marine environments [10], [11], facilitate horizontal gene transfer [12], [13], [14], and are involved in maintaining high prokaryotic richness [15], [16], [17]. Metagenomic studies have revealed that viruses carry a high proportion of unknown open reading frames [18].

In this study, we used pyro-sequencing to obtain metagenomic data on viral communities from two contrasting deep-water habitats, the Romanche Fracture Zone in the Atlantic Ocean and the southwest Mediterranean Sea. The sample from the Atlantic Ocean is representative of a typical and relatively stable deep-water habitat with little exchange of organisms and viruses between deep and surface waters leading overall to stratified prokaryotic communities [19]. However, the Mediterranean Sea is subject to regular deep vertical mixing during the non-stratified period, representing the beginning of an annual succession detectable throughout the water column. Given the specific hydrography of the Mediterranean Sea and its consequences for prokaryotes and viruses [4], [5], [6], we hypothesized that viral communities from the deep waters of the Atlantic Ocean and the Mediterranean Sea are distinctly different.

## Materials and Methods

### Ethics Statement

No specific permissions were required for the sampling locations (Table 1) and activities, and our study did not involve endangered or protected species.

**Table 1:** Details of the sampling sites where the viral metagenomes have been collected in the Atlantic Ocean and the Mediterranean Sea.

Parameter	Atlantic Ocean	Mediterranean Sea
Sampling date	5 May 2008	4 October 2003
Sample volume	130 L	500 L
Latitude	1.19° N	37.18° N
Longitude	12.96° W	14.98° E
Depth	5200 m	2400 m
Salinity	34.844	38.400
Temperature	2.04°C	13.10°C
Prokaryotic abundance	1.9×10^4^ mL^−1^	3.6×10^4^ mL^−1^
Viral abundance	4.6×10^5^ mL^−1^	9.1×10^5^ mL^−1^

### Sampling and contextual parameters

Samples were retrieved from the Romanche Fracture Zone in the Atlantic Ocean at 5200 m depth and the southwest Mediterranean Sea from 2400 m depth (Table 1) using Niskin bottles mounted on a rosette frame also holding the sensors for salinity, temperature, and depth. Sub-samples to determine prokaryotic and viral abundances were taken from untreated samples, fixed with glutaraldehyde (0.5% final concentration), flash-frozen in liquid nitrogen, and maintained at −80°C until analysis. Flow cytometric determination of prokaryotic and viral abundance was performed on a FACSCalibur (BD Biosciences) flow cytometer as previously described [20], [21].

### Obtaining metagenomic libraries and data analyses

#### Preparation of viral concentrates, DNA extraction, and sequencing

Water (Table 1) was filtered over 0.22 µm polycarbonate membrane filters (293 mm diameter; Nuclepore; Whatman) to remove cellular organisms. Subsequently, viruses in the filtrate were concentrated successively using a spiral-wound ultra-filtration device suitable for large volumes (PTHK Prep/Scale-TFF; Millipore; Atlantic Ocean: 30 kDa molecular weight cutoff [MWCO]; Mediterranean Sea: 100 kDa MWCO) followed by a smaller ultra-filtration device (Vivaflow 200; 30 kDa MWCO; PES; Sartorius Stedim Biotech) until a final volume of ∼50 mL was reached. The virus concentrates were flash-frozen in liquid nitrogen and stored at −80°C until processed. Subsequently, virus concentrates were thawed and further concentrated to a final volume of 200 µL using Amicon Ultra-15 centrifugal ultra-filtration devices (Ultracel; 30 kDa MWCO; Millipore) by repeatedly loading ∼15 mL of concentrate onto the filters until the entire volume of each concentrate was passed through one filter. DNA extraction was performed using a QIAmp MinElute Virus Spin Kit (Qiagen) following the manufacturer's instructions. Viral metagenomic data sets were obtained from at least 100 ng of DNA per sample by pyro-sequencing on 1/4 of a pico-titer plate using Roche-454 GS FLX Titanium technology performed at the Broad Institute of MIT and Harvard (Cambridge, USA).

#### Quality screening and removal of contaminating reads

Reads were quality-screened using the software PRINSEQ (version 0.20.3; [22]). First, duplicate reads were removed from the libraries. Subsequently, distinct reads were trimmed from both ends until the remaining sequences had a Phred score of at least 20 with a minimum length of 100 nt. DECONSEQ (version 0.4.3; [23]) was used to detect and remove contaminating reads with similarities to archaeal, bacterial, and eukaryotic genomes from both libraries as defined by a coverage of at least 90% and identities of at least 94%. Finally, reads composed exclusively of poly-ATCG were removed. All data presented here are solely based on reads passing these initial quality-screening steps.

#### Data analyses

To determine the degree of overlap between the two metagenomic libraries, the reads in both libraries were compared with each other using "Blast 2 Sequences" ([24]; significance at *e*-values ≤1×10^−5^). Taxonomic classification of reads and determining the relative abundance of archaeal, bacterial, and eukaryotic virus genomes were performed using the software package Genome relative Abundance and Average Size (GAAS; version 0.17; [25]) of Metavir [26] and the non-redundant viral RefSeq database (release 62; [27], [28]). The GAAS software package uses a BLAST-based [29] approach and was specifically designed to deal with a number of common problems in analyzing metagenomic data. GAAS performs length normalization to correct for sampling biases (i.e., larger genomes will have a higher number of hits compared to smaller genomes). GAAS uses similarity weighting to control for multiple BLAST hits and circumvents the problem of arbitrary thresholds for selecting significant similarities by using relative alignment lengths. The results from GAAS for the two libraries were initially visualized with the hierarchical data browser Krona ([30]; Files S1–S2). Subsequently, virus genomes found in both viromes and found only in either the Atlantic Ocean or the Mediterranean Sea were identified. Finally, virus genomes were grouped according to higher taxonomic levels (i.e., families) as given in the non-redundant viral RefSeq database. The size (i.e., the number of genomes detected per taxonomic group) and the relative abundance (calculated by summing-up the relative abundances of individual genomes) of each taxonomic group were recorded. For comparison, BLASTN searches ([29]; significance at *e*-values ≤1×10^−5^) were also performed against the environmental database (env_nt) of GenBank. Data analyses were performed between November 2013–January 2014.

#### Nucleotide sequence accession numbers

The metagenomic libraries analyzed in this study were deposited into GenBank under the accession numbers SRX042488–SRX042489 (Atlantic Ocean) and SRX042547–SRX042548 (Mediterranean Sea).

## Results

### Physical characteristics at the sampling sites and descriptive statistics of viral metagenomes

Salinity and temperature at the Mediterranean Sea sampling site were higher than at the Atlantic Ocean site (Table 1). Similarly, prokaryotic and viral abundances were about twice as high in the Mediterranean Sea than in the Atlantic Ocean sample (Table 1). We obtained 90,174 reads from the viral size fraction of the Atlantic Ocean of which 82,792 reads passed the quality criteria (Table 2). The sample from the Mediterranean Sea yielded 104,669 reads, with 44,974 reads passing the quality screening (Table 2). The average read length in the sample of the Atlantic Ocean and the Mediterranean Sea was 411 bp and 370 bp, respectively. Based on a threshold of 90% sequence similarity, the richness (i.e., number of distinct reads) of the Atlantic Ocean virome was higher than that of the Mediterranean Sea (Fig. S1). Only 2.6% (3,282 reads) of all reads were found in both libraries. The average GC content was 42.8% in the Atlantic Ocean sample and 47.8% in the Mediterranean Sea sample (Table 2). On average, the GC content was significantly lower in the Atlantic Ocean sample than in the Mediterranean Sea (Mann-Whitney test: *U* = 1.4×10^7^, *p*<0.0001).

**Table 2:** Basic characteristics of the viral metagenomes obtained from the Atlantic Ocean and the Mediterranean Sea.

Parameter	Atlantic Ocean	Mediterranean Sea
Total sequence length	34.04×10^6^ bp	16.62×10^6^ bp
Number of reads	82,792	44,974
Average length	411 bp	370 bp
Minimum length	100 bp	100 bp
Maximum length	678 bp	633 bp
GC content	42.8%	47.8%

### Comparison of Atlantic Ocean and Mediterranean Sea viromes

#### Number and relative abundance of specific virus groups

**Figure 1:**
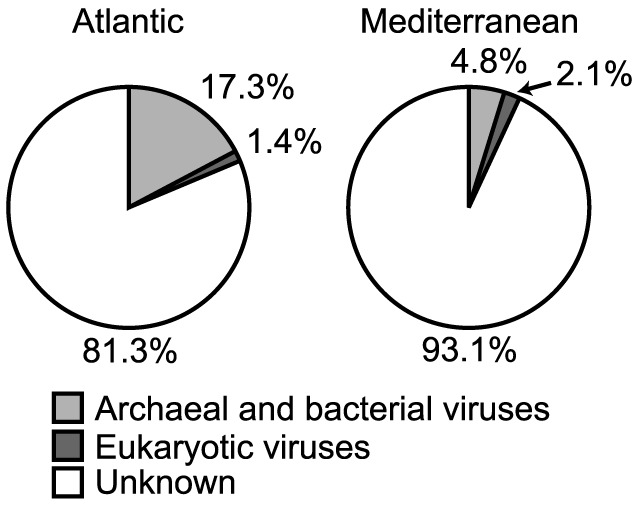
Percentage of hits in the non-redundant viral RefSeq database. The figure shows the percentage of hits (% of total reads) to archaeal and bacterial as well as to eukaryotic virus genomes in the non-redundant viral RefSeq database for the Atlantic Ocean and Mediterranean Sea viromes.

For the Atlantic Ocean virome, we found 14,286 reads (17.3% of reads) with hits to archaeal and bacterial virus genomes, 1,176 reads (1.4% of reads) with hits to eukaryotic virus genomes, and 67,330 reads (81.3%) had no hits in the non-redundant viral RefSeq database (Fig. 1). The analysis of the Mediterranean sea virome resulted in 2,149 reads (4.8% of reads) with similarities to archaeal and bacterial virus genomes, 961 reads (2.1% of reads) were grouped with genomes of eukaryotic viruses, and 41,864 reads (93.1% of reads) were not related to any of the genomes in the non-redundant viral RefSeq database (Fig. 1). In comparison, BLASTN searches revealed substantially more reads with significant similarities to sequences in the env_nt database of GenBank (Table S1: Atlantic Ocean: 42% of reads, Mediterranean Sea: 48% of reads). Reads from the Atlantic Ocean virome had significant hits to 786 distinct archaeal and bacterial virus genomes and 85 eukaryotic virus genomes (871 genomes in total), whereas reads from the Mediterranean Sea virome grouped with 432 archaeal and bacterial virus genomes and 83 eukaryotic virus genomes (515 genomes in total). In total, the reads from both viromes had significant hits to 946 distinct genomes (828 archaeal and bacterial virus genomes, 118 eukaryotic virus genomes) contained in the non-redundant viral RefSeq database. Of these 946 genomes, 440 genomes were shared by both viromes.

**Figure 2:**
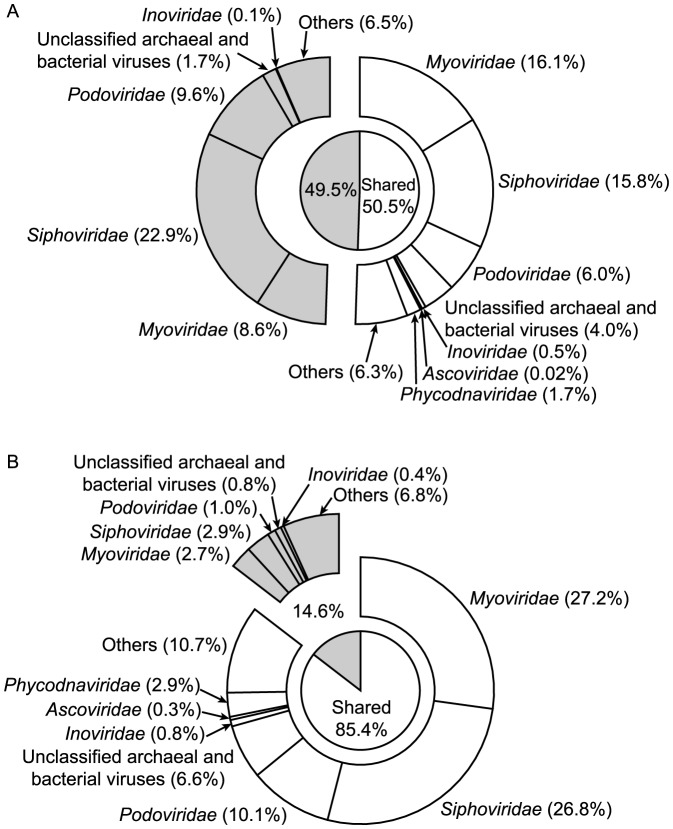
Size of specific virus groups. The figure shows the size of specific virus families in terms of the number of genomes expressed as percentage of the total number of genomes detected in (A) the Atlantic Ocean (*N* = 871) and (B) the Mediterranean Sea (*N* = 515). Additionally, the light area represents the contribution of genomes found in both viromes whereas the shaded area depicts genomes found exclusively in either (A) the Atlantic Ocean or (B) the Mediterranean Sea. No unique *Ascoviridae* and *Phycodnaviridae* could be identified. A list of virus groups found in "Others" given in Table S2.

**Figure 3:**
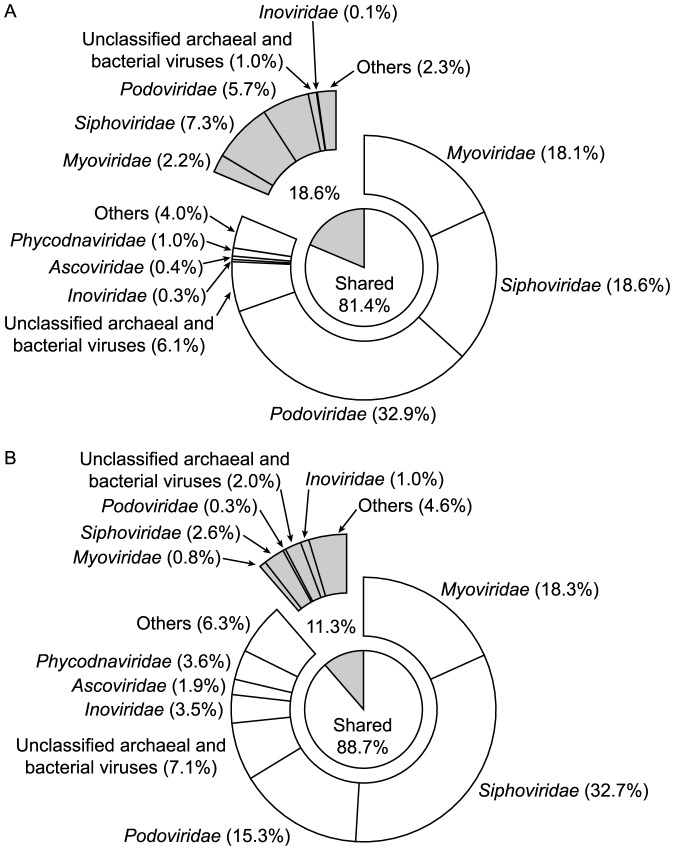
Relative abundance of specific virus groups. The figure shows the relative abundance of specific virus families as calculated by adding the relative abundance of individual virus genomes for (A) the Atlantic Ocean and (B) the Mediterranean Sea. Additionally, the light area represents the relative abundance of virus groups composed of virus genomes found in both viromes whereas the shaded area depicts the relative abundance of virus groups found exclusively in either (A) the Atlantic Ocean or (B) the Mediterranean Sea. No unique *Ascoviridae* and *Phycodnaviridae* could be identified. A list of virus groups found in "Others" given in Table S2.

Virus genomes found in both viromes contributed 50.5% of the total number of genomes detected in the Atlantic Ocean and 85.4% in the Mediterranean Sea (Fig. 2). In both viromes and for the shared and unique groups of viruses, archaeal and bacterial viruses together (*Myoviridae*, *Siphoviridae*, *Podoviridae*, unclassified archaeal and bacterial viruses, *Inoviridae*) contributed the largest number of genomes collectively accounting for 85.3% of the genomes in the Atlantic Ocean (Fig. 2A; 42.4% and 42.9% in the shared and unique virus communities, respectively) and for 79.3% in the Mediterranean Sea (Fig. 2B; 71.5% and 7.8% in the shared and unique virus communities, respectively). Despite the substantial difference in the contribution of the 440 shared genomes to the total number of identifiable genomes in the Atlantic Ocean and the Mediterranean Sea (Fig. 2), the relative abundance of the shared genomes was surprisingly similar in both viromes accounting for 81.4% in the Atlantic Ocean and for 88.7% in the Mediterranean Sea (Fig. 3). Archaeal and bacterial viruses were numerically dominant, together accounting for 92.3% of the relative abundance in the Atlantic Ocean (Fig. 3A; 76.0% and 16.3% in the shared and unique virus communities, respectively) and for 83.6% in the Mediterranean Sea (Fig. 3B; 76.9% and 6.7% in the shared and unique virus communities, respectively). Focusing only on the shared virus genomes, the dominant group of viruses in the Atlantic Ocean was *Podoviridae* (Fig. 3A; 32.9%), although only accounting for 6.0% (Fig. 2A) of identifiable virus genomes in the same virome. In contrast, the virus community comprised of shared viruses in the Mediterranean Sea was dominated by *Siphoviridae* (Fig. 3B; 32.7%), also constituting one of the largest groups in terms of the number of genomes in that virome (Fig. 2B; 26.8%). Additionally, in the Mediterranean Sea (Fig. S2B) the size of the virus groups (Fig. 2B) was positively related to the corresponding relative abundance (Fig. 3B), whereas such a relationship was not found for the Atlantic Ocean (Fig. S2A; Figs. 2A and 3A).

The rank-abundance curves for both viromes were similar and characterized by a small number of abundant genomes and a large number of genomes with a relative abundance <1% (Fig. S3). Despite these similarities, the most abundant individual virus genomes differed between the two viromes. In the Atlantic Ocean, the five most abundant virus genomes were identified as Pelagibacter phage HTVC010P (8.9%), Puniceispirillum phage HMO-2011 (7.6%), Pelagibacter phage HTVC019P (2.8%), and Pelagibacter phage HTVC011P (2.4%) all belonging to the family *Podoviridae* and a member of the *Myoviridae*, Pelagibacter phage HTVC008M (4.6%; File S1). In contrast, the most abundant virus genomes in the Mediterranean Sea were identified as Planktothrix phage PaV-LD (4.0%) and Pelagibacter phage HTVC010P, both belonging to the family *Podoviridae*, Lactococcus phage bIL312 (3.2%; *Siphoviridae*), Groundnut rosette virus satellite RNA (2.6%; satellite nucleic acids), and Enterobacteria phage I2-2 (1.9%, *Inoviridae*; File S2).

#### GC content

Overall, the GC content for both viromes showed a bimodal distribution (Fig. S4A–B). In the Atlantic Ocean virome, the GC content exhibited a pronounced peak at 36.5% and a smaller one at 53.5% (Fig. S4A). In the Mediterranean Sea virome, the GC content had a maximum at 40.5% and a second one at 62.5% (Fig. S4B). For both viromes, the GC content distribution of reads with similarities to the genomes of archaeal and bacterial viruses (Fig. S4C–D) and eukaryotic viruses (Fig. S4E–F) was comparable to the distribution found when analyzing all reads in the respective libraries.

## Discussion

### A common core set of viruses

Although only 2.6% of the reads were shared by both viromes, a total of 440 virus genomes were common to the Atlantic Ocean and the Mediterranean Sea. The contribution of the number of shared virus genomes to the total number of identified genomes differed strongly between the Atlantic Ocean (50.5%) and the Mediterranean Sea (85.4%; Fig. 2). However, commonly occurring viruses accounted for 81.4% and 88.7% of the relative abundance in the Atlantic Ocean and the Mediterranean Sea, respectively (Fig. 3). Thus, although the number of virus types uniquely found in the Atlantic Ocean (431 unique virus genomes) was much higher compared to the Mediterranean Sea (75 unique virus genomes), their contribution to the overall relative abundance was small and comparable in both viromes (Fig. 3). These results suggest that the contrasting deep-sea environments of the Atlantic Ocean and the Mediterranean Sea share a common core set of virus types (Fig. 2) that numerically dominate both virus communities with most of the shared virus types infecting prokaryotes (Atlantic Ocean: 76.0%; Mediterranean Sea: 76.9%).

### The dominance of archaeal and bacterial viruses

The Atlantic Ocean virome exhibited a higher richness (i.e., a higher number of distinct reads) than the Mediterranean Sea virome (Fig. S1). The higher number of distinct reads in the sample from the Atlantic Ocean compared to the Mediterranean Sea also translated into a higher number of distinct virus genomes (Atlantic Ocean: 871 genomes in total; Mediterranean Sea: 515 genomes in total). However, both viromes were dominated by archaeal and bacterial viruses in terms of the number of identifiable virus genomes (Atlantic Ocean: 85.3%; Mediterranean Sea: 79.3%; Fig. 2) and their relative abundance (Atlantic Ocean: 92.3%; Mediterranean Sea: 83.6%; Fig. 3). Given that prokaryotes are the most abundant group of organisms in the ocean, especially in the deep [31], and that viruses depend entirely on their hosts for proliferation, these results confirm the notion that the majority of viruses found in the ocean infects prokaryotes. This finding is not new, however, it does serve as an additional sanity check for the analyses.

### The "rare virosphere"

Prokaryotic and viral communities in deep waters of the Mediterranean Sea exhibit seasonality [4], [5], [6]. Consequently, the results from comparing metagenomic data on deep-water virus communities between the Atlantic Ocean and the Mediterranean Sea are likely to vary seasonally. Nevertheless, the relative abundance of genomes in both viromes appears to follow the same principle with a small number of genomes dominating the community and a large number of genomes present in low relative abundances (Fig. S2). These results are similar to the composition of deep-sea bacterial communities as detected by massively parallel tag-sequencing [19], [32] and suggest the existence of a "rare virosphere" in deep waters of the Atlantic Ocean and the Mediterranean Sea.

### The influence of hydrography-attempting an ecological explanation

In the Mediterranean Sea, the relative abundance of virus groups was significantly positively related to the number of virus genomes in these virus groups (Fig. S2B), whereas such a relationship was not found in the Atlantic Ocean (Fig. S2A). These results suggest that in the Atlantic Ocean virome, the relative abundance of a specific virus group is not only driven by its size, i.e., the number of virus genomes in that group, but by a process that selectively favors certain virus groups. Obviously, any pattern identified for virus communities will be related to their host communities, that mostly consist of prokaryotes at depth [31]. Bacterial communities in deep Atlantic Ocean waters are depth-stratified [19], with specific water masses harboring distinctly different bacterial communities. A stratified water column constitutes a stable environment, allowing certain groups of prokaryotes to outcompete other, less adapted types. In contrast, prokaryotic communities found in deep waters of the Mediterranean Sea have been shown to be seasonally as dynamic as surface water communities [4], [5], [6] due to the specific hydrography of the Mediterranean, allowing deep vertical mixing driven by storms during the non-stratified period [3] and thereby eroding any detectable stratification in prokaryotic communities. The sample in the Mediterranean Sea was taken in October (Table 1), i.e., at the onset of the non-stratified period. Thus, the linear relationship between the size of specific virus groups and their relative abundance (Fig. S2B) in the Mediterranean Sea may be the result of a more uniform prokaryotic host community, potentially driven by deep vertical mixing during the non-stratified period. The higher number (Fig. 2) and relative abundance (Fig. 3) of detected eukaryotic virus genomes (*Ascoviridae*, *Phycodnaviridae*, and virus genomes grouped into "Others") in the Mediterranean Sea compared to the Atlantic Ocean suggest that some import of eukaryotic viruses may already have occurred. In contrast, the stratified prokaryotic host community in the Atlantic Ocean, well adapted to a specific water mass with little physical disturbance due to mixing, may have lead to the observed dominance in relative abundance of specific virus groups (e.g., *Siphoviridae*, *Podoviridae*; Figs. 2A and 3A), disproportionate to their contribution to overall richness (Fig. S2A).

### Methodological considerations

Although the sample volume for the Mediterranean Sea virome was 3.8-times larger and viral abundance was twice as high compared to the Atlantic Ocean (Table 1), we found 1.8-times more distinct archaeal and bacterial virus genomes in the Atlantic Ocean (786 archaeal and bacterial virus genomes) compared to the Mediterranean Sea virome (432 archaeal and bacterial virus genomes). However, the number of distinct eukaryotic virus genomes was similar in both samples (Atlantic Ocean: 85 eukaryotic virus genomes; Mediterranean Sea: 83 eukaryotic virus genomes). In principle, this difference might be due to the different molecular weight cut-off (MWCO) of the filtration devices used to obtain the Atlantic Ocean (30 kDa MWCO) and Mediterranean Sea (100 kDa MWCO) virus concentrates. However, the equivalent pore size of a filtration device with a MWCO of 30 kDa is 4 nm and a MWCO of 100 kDa corresponds to a pore size of 6 nm [33]. Thus, the pore size of both filtration devices was at least one order of magnitude lower than the size of marine archaeal and bacterial viruses and differed only by 2 nm. Additionally, the filtrates from both filtration devices were inspected using flow cytometry on multiple occasions and did not reveal the presence of viruses using SYBR Green I-staining [21]. Thus, it is unlikely that the difference in the number of distinct archaeal and bacterial virus genomes identifiable between the Atlantic Ocean and the Mediterranean Sea sample is attributable to sampling issues.

### Advantages and pitfalls of curated reference databases

In this study the analysis strategy was to align relatively short DNA sequences with whole genome sequences of viruses (viral non-redundant RefSeq database; [27], [28]). The advantage of this approach lies in a reduction of dimensionality; many relatively short DNA reads are grouped into fewer and longer entities that are easier to compare and interpret. However, the amount of high-quality, curated data in reference databases is necessarily much smaller than large data repositories such as env_nt of GenBank. Thus, it is not surprising that a comparison between the number of hits obtained against the non-redundant viral RefSeq database (Fig. 1) with the number of hits obtained by BLASTN against env_nt of GenBank (Table S1) revealed that 18.7% and 6.9% of reads from the Atlantic Ocean and Mediterranean Sea virome, respectively, had similarities in RefSeq whereas 42% of reads from the Atlantic Ocean and 48% of reads from the Mediterranean Sea were similar to sequences in env_nt. Also, a closer inspection of the types of viruses contained in RefSeq with hits to reads from either virome (Files S1–S2) suggests that the exact phylogenetic identities of the detected genomes have to be interpreted cautiously. For example, viruses infecting the cyanobacterial genera *Prochlorococcus* and *Synechococcus* certainly occur in marine environments, although not necessarily in high abundances at the depths we retrieved our samples (Table 1). It has also been shown that relatives of Mimivirus (infecting the amoeba *Acanthamoeba polyphaga*) are widespread in marine environments [34]. However, for a number of viruses, it is difficult to imagine how they could occur in samples from the deep-sea (e.g., Groundnut rosette virus satellite RNA). Additionally, archaeal and bacterial virus sequences may be incorrectly annotated as prokaryotic chromosomal sequences when, in reality, they represent unrecognized prophages or cryptic phage fragment regions [35]. Moreover, as archaeal and bacterial virus genomes are often constructed in modules, detected homologies to relatively short metagenomic reads may be of limited use for accurately identifying archaeal and bacterial viruses [36]. Thus, the results from the non-redundant viral RefSeq database should not be taken as definite phylogenetic identification of the types of viruses present in the samples. Instead, the results might indicate the presence of related viruses that are currently not in the RefSeq database. Because of these uncertainties, we have restricted the presentation and discussion of the data to higher taxonomic levels, i.e., virus families (Figs. 2–3), for which taxonomic classifications are more reliable.

Unfortunately, the data and consequently the conclusions drawn from comparing metagenomic datasets with each other may not only be influenced by low and variable hit rates of reads against reference databases (Fig. 1, Table S1). Other factors possibly influencing the results as well, including differences in the amount of reads passing the quality control and the part of the richness in the samples not covered (Fig. S1). All of these issues are inherent to comparative metagenomic studies. Thus, the identifiable richness of deep-sea virus communities from the Atlantic Ocean and the Mediterranean Sea will likely increase with the size of the non-redundant viral RefSeq database and sequencing effort. However, the identification of a common core set of virus types, accounting for a large and comparable part of the relative abundance in both viromes constitutes a pattern that is unlikely to change dramatically, considering the large differences in richness versus relative abundance of common and unique viruses between the Atlantic Ocean and the Mediterranean Sea viromes (Figs. 2–3).

## Summary and Conclusions

The results and conclusion are based on 15,462 reads (18.7%) and 3,110 reads (6.9%; Fig. 1) obtained from the Atlantic Ocean and the Mediterranean Sea sample, respectively, with significant similarities to virus genomes in the non-redundant viral RefSeq database. Both viromes were dominated by archaeal and bacterial viruses in terms of the number of identifiable virus genomes (Atlantic Ocean: 85.3%; Mediterranean Sea: 79.3%; Fig. 2) and their relative abundance (Atlantic Ocean: 92.3%; Mediterranean Sea: 83.6%; Fig. 3). Initially, we hypothesized that the viral communities from the deep waters of the Atlantic Ocean and the Mediterranean Sea would be distinctly different from each other. However, the results suggest that the contrasting deep-sea environments of the Atlantic Ocean and the Mediterranean Sea share a common core set of virus types (440 virus genomes), that dominate both virus communities in terms of relative abundance (Atlantic Ocean: 81.4%; Mediterranean Sea: 88.7%), independent of additionally found virus genomes unique to either the Atlantic Ocean (431 virus genomes) or the Mediterranean Sea (75 virus genomes).

## Supporting Information

Figure S1 **Rarefaction curves.** The figure depicts the rarefaction curves of the Atlantic Ocean and Mediterranean Sea viromes based on a sequence similarity threshold of 90%.
(PDF)

Figure S2 **Relationship between size and relative abundance of virus taxonomic groups.** In this figure, the size of virus taxonomic groups (Fig. 2) were plotted against the respective relative abundance (Fig. 3). The solid line represents the linear least-squares regression calculated between the size and relative abundance of virus taxonomic groups in (A) the Atlantic Ocean (*y* = 0.65 *x*+2.17, *r^2^* = 0.24, *p* = 0.0557, *N* = 16) and (B) the Mediterranean Sea (*y* = 0.92 *x*+0.49, *r^2^* = 0.85, *p*<0.0001, *N* = 16). Dashed lines represent the one-to-one line.
(PDF)

Figure S3 **Rank abundance curves.** The figure shows the rank-abundance curves for virus genomes contained in the non-redundant viral RefSeq database to which reads from the Atlantic Ocean and Mediterranean Sea viromes had significant hits.
(PDF)

Figure S4 **GC content.** The figure depicts histograms (binning size 1 bp) for reads of the (A) Atlantic Ocean and (B) Mediterranean Sea libraries. Additionally, the GC content is shown for reads having significant similarities to archaeal and bacterial or eukaryotic virus genomes of the Atlantic Ocean (C, E) and Mediterranean Sea viromes (D, F).
(PDF)

Table S1 **Comparison of reads against the environmental database (env_nt) of GenBank.** The table shows the number and percentage of reads with significant hits and no homology to sequences in the env_nt database of GenBank as determined by BLASTN.
(DOC)

Table S2 **Taxonomic groups collectively referred to as "Others".** The table gives a list of taxonomic groups from the non-redundant viral RefSeq database that have been referred to as "Others" in Figs. 2–3.
(DOC)

File S1 **Interactive pie chart of the relative abundance of genomes as detected in the Atlantic Ocean virome.** The file opens in a web browser and shows the relative abundance and phylogenetic affiliation of the genomes detected in the Atlantic Ocean virome.
(HTML)

File S2 **Interactive pie chart of the relative abundance of genomes as detected in the Mediterranean Sea virome.** The file opens in a web browser and shows the relative abundance and phylogenetic affiliation of the genomes detected in the Mediterranean Sea virome.
(HTML)
