# Supplementary figures and images for: Comparison of Deep-Water Viromes from the Atlantic Ocean and the Mediterranean Sea
Source: PLoS One. 2014 Jun 24;9(6):e100600. doi: 10.1371/journal.pone.0100600 (PMC4069082; doi:10.1371/journal.pone.0100600)

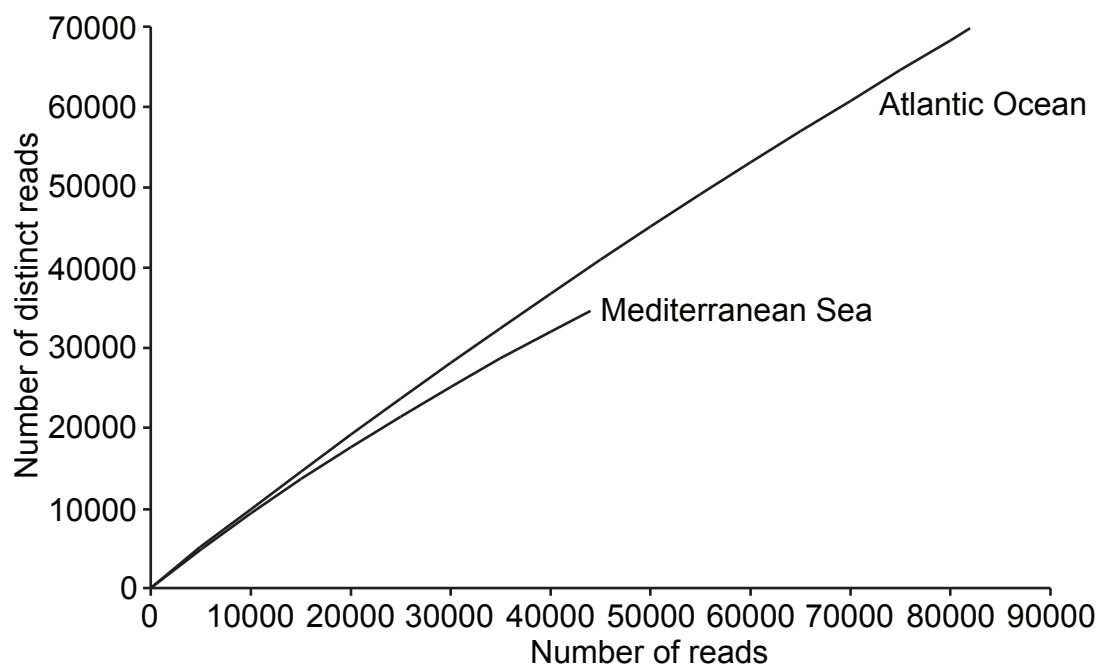

Supplement: Figure S1 — Rarefaction curves. The figure depicts the rarefaction curves of the Atlantic Ocean and Mediterranean Sea viromes based on a sequence similarity threshold of 90%. (PDF) [file pone.0100600.s001.pdf]

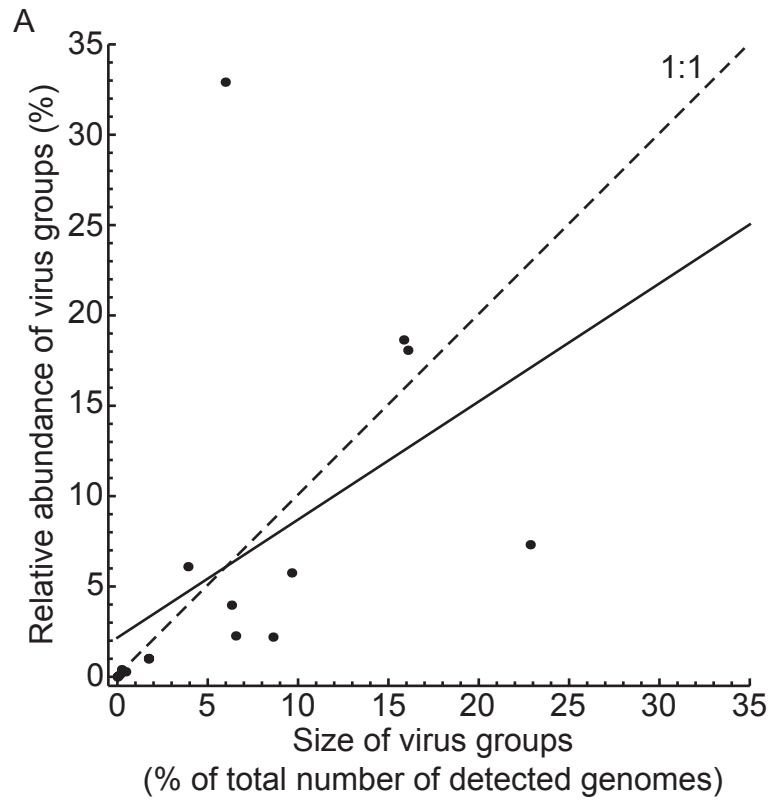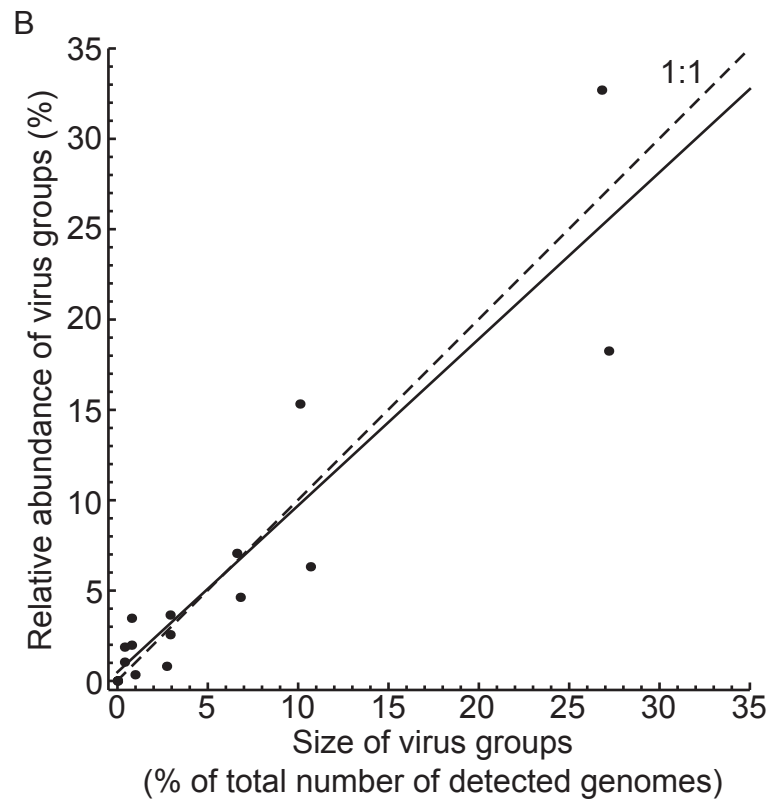

Supplement: Figure S2 — Relationship between size and relative abundance of virus taxonomic groups. In this figure, the size of virus taxonomic groups (Fig. 2) were plotted against the respective relative abundance (Fig. 3). The solid line represents the linear least-squares regression calculated between the size and relative abundance of virus taxonomic groups in (A) the Atlantic Ocean (y = 0.65 x+2.17, r2 = 0.24, p = 0.0557, N = 16) and (B) the Mediterranean Sea (y = 0.92 x+0.49, r2 = 0.85, p<0.0001, N = 16). Dashed lines represent the one-to-one line. (PDF) [file pone.0100600.s002.pdf]

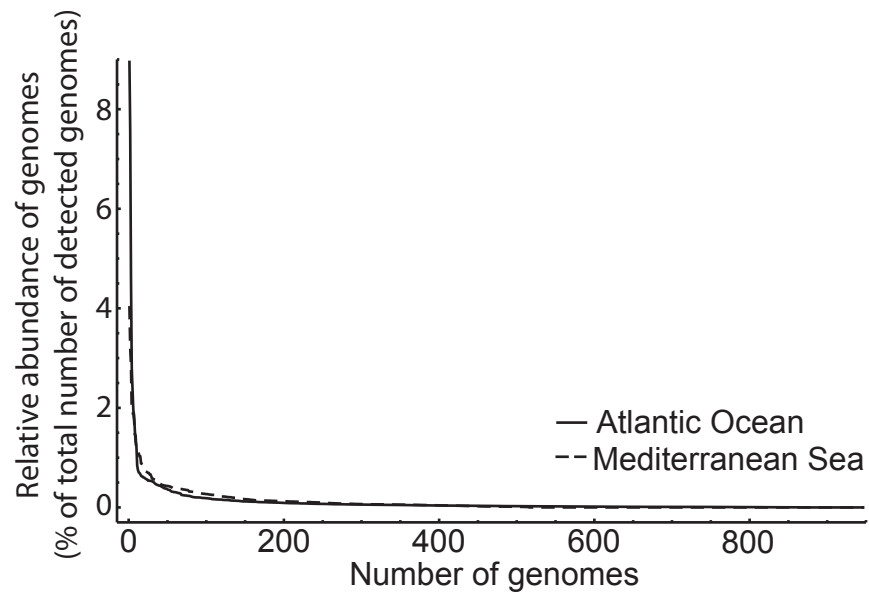

Supplement: Figure S3 — Rank abundance curves. The figure shows the rank-abundance curves for virus genomes contained in the non-redundant viral RefSeq database to which reads from the Atlantic Ocean and Mediterranean Sea viromes had significant hits. (PDF) [file pone.0100600.s003.pdf]

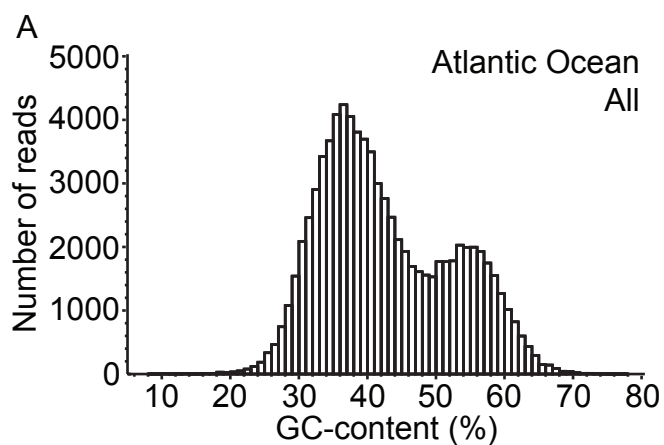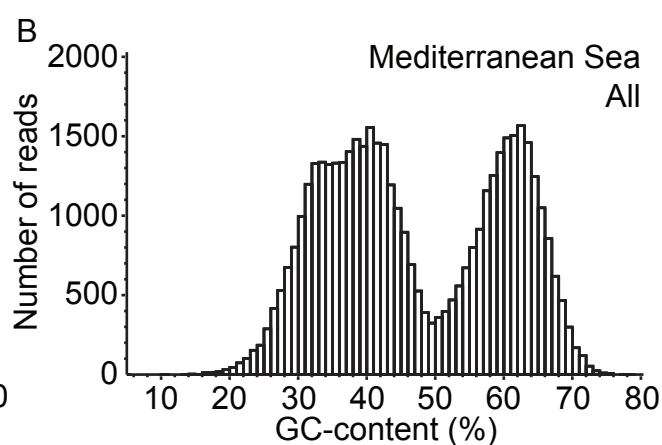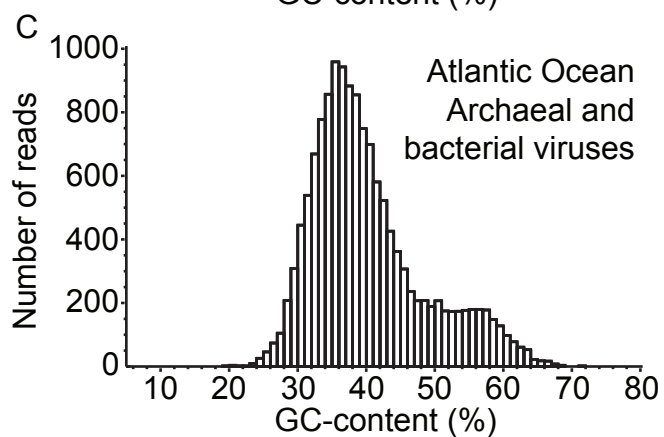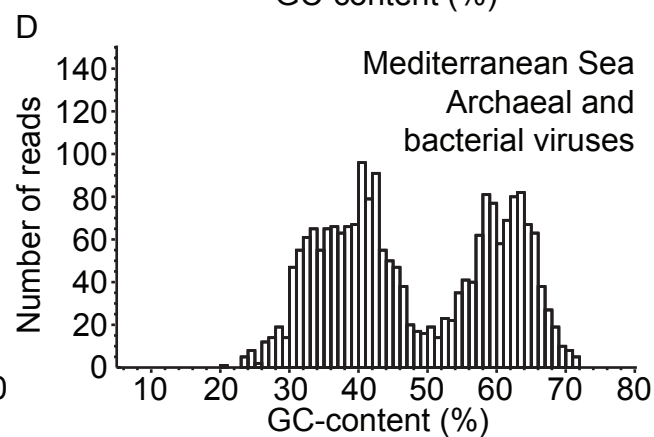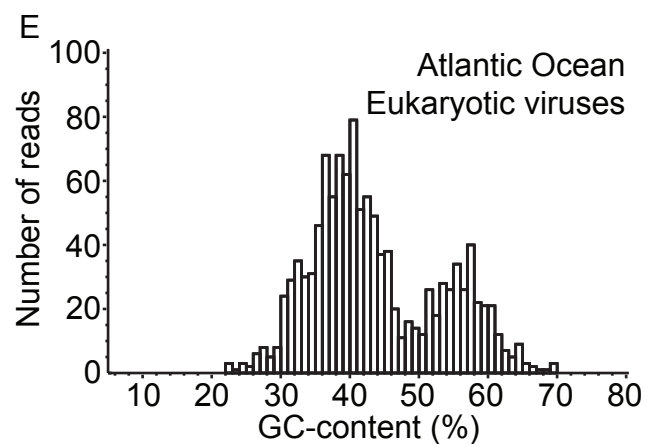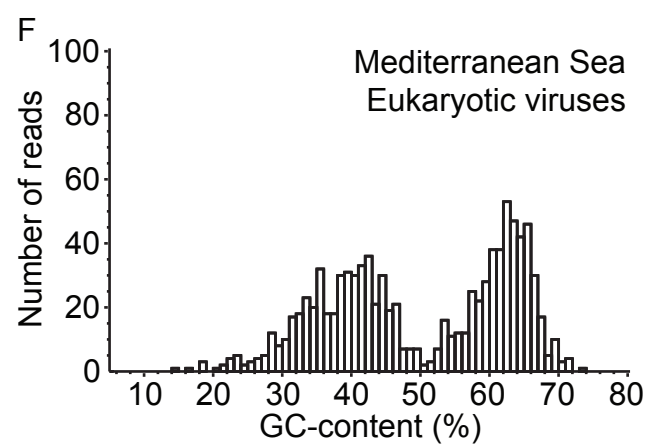

Supplement: Figure S4 — GC content. The figure depicts histograms (binning size 1 bp) for reads of the (A) Atlantic Ocean and (B) Mediterranean Sea libraries. Additionally, the GC content is shown for reads having significant similarities to archaeal and bacterial or eukaryotic virus genomes of the Atlantic Ocean (C, E) and Mediterranean Sea viromes (D, F). (PDF) [file pone.0100600.s004.pdf]
