# Supplementary material for: Comparison of Deep-Water Viromes from the Atlantic Ocean and the Mediterranean Sea
Source: PLoS One. 2014 Jun 24;9(6):e100600. doi: 10.1371/journal.pone.0100600 (PMC4069082; doi:10.1371/journal.pone.0100600)
Supplement: Table S1 — Comparison of reads against the environmental database (env_nt) of GenBank. The table shows the number and percentage of reads with significant hits and no homology to sequences in the env_nt database of GenBank as determined by BLASTN. (DOC) [file pone.0100600.s005.doc]

| BLASTN against env_nt | Atlantic Ocean | | Mediterranean Sea | |
| --- | --- | --- | --- | --- |
|  | Number | % of reads | Number | % of reads |
| Reads with hits | 34,954 | 42 | 21,451 | 48 |
| No homology | 47,838 | 58 | 23,523 | 52 |
| Total | 82,792 | 100 | 44,974 | 100 |
