# Supplementary material for: Comparison of Deep-Water Viromes from the Atlantic Ocean and the Mediterranean Sea
Source: PLoS One. 2014 Jun 24;9(6):e100600. doi: 10.1371/journal.pone.0100600 (PMC4069082; doi:10.1371/journal.pone.0100600)
Supplement: Table S2 — Taxonomic groups collectively referred to as "Others". The table gives a list of taxonomic groups from the non-redundant viral RefSeq database that have been referred to as "Others" in Figs. 2–3. (DOC) [file pone.0100600.s006.doc]

| Taxonomic groups in "Others" | Atlantic Ocean | Mediterranean Sea |
| --- | --- | --- |
| *Asfarviridae* | + | - |
| *Baculoviridae* | + | + |
| *Bicaudaviridae* | + | + |
| *Corticoviridae* | + | + |
| *Fuselloviridae* | - | + |
| *Herpesviridae* | + | + |
| *Alloherpesviridae* | + | + |
| *Iridoviridae* | + | + |
| *Lipothrixviridae* | + | + |
| *Mimiviridae* | + | + |
| *Polydnaviridae* | + | - |
| *Poxviridae* | + | + |
| *Rudiviridae* | + | - |
| *Tectiviridae* | + | + |
| unclassified dsDNA viruses | + | + |
| *Retroviridae* | + | + |
| Satellite nucleic acids | - | + |
| *Mononegavirales* | + | - |
| *Potyviridae* | + | + |
| *Virgaviridae* | + | - |
| Unclassified virophages | + | + |
| Unclassified viruses | + | + |
