# Supplementary material for: Comparison of Deep-Water Viromes from the Atlantic Ocean and the Mediterranean Sea
Source: PLoS One. 2014 Jun 24;9(6):e100600. doi: 10.1371/journal.pone.0100600 (PMC4069082; doi:10.1371/journal.pone.0100600)
Supplement: File S1 — Interactive pie chart of the relative abundance of genomes as detected in the Atlantic Ocean virome. The file opens in a web browser and shows the relative abundance and phylogenetic affiliation of the genomes detected in the Atlantic Ocean virome. (HTML) [file pone.0100600.s007.html]

This browser does not support HTML5 (see
Krona browser support).
